# Emerging Role and Mechanism of circRNAs in Pediatric Malignant Solid Tumors

**DOI:** 10.3389/fgene.2021.820936

**Published:** 2022-01-18

**Authors:** Qiyang Shen, Xingyu Liu, Wei Li, Xu Zhao, Tao Li, Kai Zhou, Jianfeng Zhou

**Affiliations:** ^1^ Department of Pediatric Surgery, Children’s Hospital of Nanjing Medical University, Nanjing, China; ^2^ Department of Pediatric Surgery, First Affiliated Hospital of Bengbu Medical College, Bengbu, China; ^3^ Department of ENT, Children’s Hospital of Nanjing Medical University, Nanjing, China

**Keywords:** pediatric malignant solid tumors, circRNA, epigenetics, biomarkers, biological functions

## Abstract

Circular RNAs (circRNAs) are non-coding RNAs with covalent closed-loop structures and are widely distributed in eukaryotes, conserved and stable as well as tissue-specific. Malignant solid tumors pose a serious health risk to children and are one of the leading causes of pediatric mortality. Studies have shown that circRNAs play an important regulatory role in the development of childhood malignant solid tumors, hence are potential biomarkers and therapeutic targets for tumors. This paper reviews the biological characteristics and functions of circRNAs as well as the research progress related to childhood malignant solid tumors.

## Introduction

Although rare, childhood malignancies are now the second leading cause of pediatric death (Ollauri-Ibanez and Astigarraga, 2021; Quamine et al., 2021). Furthermore, the incidence of childhood malignancies is increasing worldwide, with less than 40% of children receiving an appropriate diagnosis and treatment (Oyefiade et al., 2021; Sze, 2021). The symptoms of childhood malignancies are often similar to those of other common, benign diseases, so early and accurate diagnosis is difficult, with an untimely diagnosis an important cause of delayed treatment and mortality (Van Paemel et al., 2020; Jain et al., 2021; Lucas et al., 2021). Although significant progress has been made in the treatment of childhood malignancies in recent years, tumor treatment based on surgery and radiotherapy can only kill tumor cells, not effectively control the recurrence and metastasis of some childhood tumors (Weiser et al., 2019; Casey and Cheung, 2020; Cimini et al., 2020; Fair et al., 2020; Greenbaum et al., 2020). Therefore, exploring early diagnostic markers of pediatric tumors to find possible therapeutic targets is important to improve the early diagnosis, treatment, and prognosis of pediatric malignant solid tumors.

Circular RNAs (circRNAs) are a class of non-coding RNAs (ncRNAs) formed by the 3 and 5′ ends of mRNAs, which are mainly generated from introns or exons by reverse splicing or lassoing introns (Ebbesen et al., 2017; Huang and Zhu, 2021). Previously, circRNAs were considered as error products in post-transcriptional processing with no important regulatory role in biological processes. However, with the development of sequencing technology, researchers have found that circRNAs are widely distributed in eukaryotic cells and can be stably expressed, playing an important role in the regulation of gene expression in human cells (Qu et al., 2017; Han et al., 2021). An increasing number of studies have shown that circRNAs have important physiopathological functions and are involved in the regulation of cell proliferation, differentiation, and apoptosis, as well as in the development of various diseases including tumors (Altesha et al., 2019; Li Y. et al., 2020; Wu et al., 2020; Xu et al., 2020; Choudhary et al., 2021; Tian et al., 2021; Zhang et al., 2021). For example, circPRKAR1B regulates FZD4 expression by binding miR-361-3p, promoting the proliferation and migration of osteosarcoma cells as well as tumor susceptibility to chemotherapy resistance (Feng Z.-h. et al., 2021). Circ_0004296 downregulates ETS1 expression by promoting retention of EIF4A3 in the nucleus and inhibiting the nuclear export of ETS1 mRNA, which in turn keeps PCa from malignant growth and metastasis (Mao S. et al., 2021). CircGSK3B directly binds to EZH2 and inhibits the binding of EZH2 and H3K27me3 to the RORA promoter, leading to elevated RORA expression and inhibiting tumor progression by suppressing the growth, invasion, and metastasis of gastric cancer cells (Ma X. et al., 2021).

Recently, circRNAs have been found to have important biological roles in childhood malignant solid tumors, hence the potential to become tumor markers and therapeutic targets. This paper reviews the formation and biological functions of circRNAs, as well as the research on circRNAs in childhood malignant solid tumors to provide new perspectives for the clinical diagnosis and treatment of childhood malignant solid tumors.

## Overview and Biological Properties of Circular RNA

**FIGURE 1:**
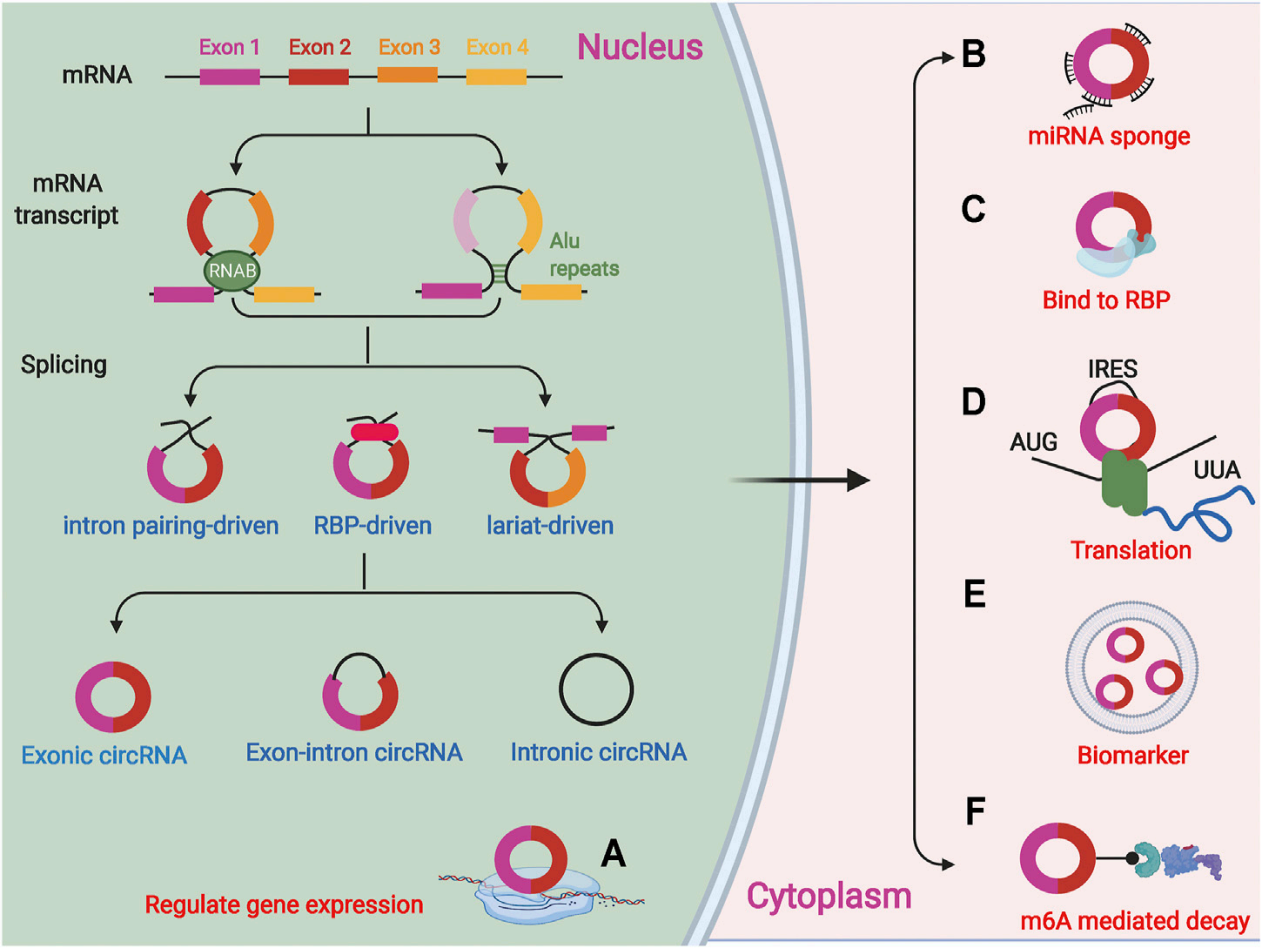
CircRNAs are a covalently closed loops formed by splicing of precursor mRNA (pre-mRNAs). There are three forms of circRNAs:lasso-driven cyclization, intron pairing cyclization and RNA binding proteins (RBPs) mediated cyclization. CircRNAs are classified into three main types, exonic circRNAs, intronic circRNAs, and exon-intron circRNAs. **(A)** CircRNAs can regulate the expression of related genes in cells; **(B)** CircRNAs as miRNA sponge can adsorb related miRNAs; **(C)** The functions of circRNA in interacting with proteins; **(D)** Several circRNAs have also been reported to encode proteins; **(E)** CircRNAs in exosomes or microvesicles can be used as specific biomarkers; **(F)** CircRNAs can be degraded via m6A-mediated decay.

CircRNAs are formed by splicing of precursor mRNAs (pre-mRNAs) and are mostly endogenous. They have no 3′ tail or 5′ cap end and are covalently closed loops (Qu et al., 2015; Meng et al., 2017) formed by lasso-driven cyclization, direct reverse splicing, or exon skipping, or they can be cyclized by intron pairing (Wang et al., 2021e; Xiao et al., 2021). In addition, circRNAs can also be formed by RNA binding proteins (RBPs)-mediated cyclization joining the downstream 5′ end donor site to the upstream 3′ end acceptor site to form a single-stranded covalent closed-loop that joins the remaining sequence after removal of the intron (Ali et al., 2021; Wang et al., 2021d). Thus, the formation of circRNAs requires the mini-intron at the splice site and the short-chain reverse repeat. Depending on the formation mechanism, circRNAs are classified into three main types, exonic circRNAs (EcircRNAs), intronic circRNAs (ciRNAs), and exon-intron circRNAs (EIciRNAs) (Lyu and Huang, 2017; Meng et al., 2017), of which, EcircRNAs are the most abundant and located in the cytoplasm of eukaryotes (Figure 1).

CircRNAs are diverse with more than 25,000 circRNAs detected in human fibroblasts by high-throughput sequencing, and in some cases, circRNA expression even exceeds that of their corresponding linear mRNAs by more than 10-fold (Shen et al., 2021; van Zonneveld et al., 2021). In contrast to mRNAs, circRNAs exist in a covalent closed-loop structure with no cap and tail structure. This unique structure makes circRNAs highly stable and resistant to hydrolysis by RNA exonucleases (Li H. M. et al., 2019), as well as consistently and stably expressed in cells (Wu et al., 2020; Shen et al., 2021). CircRNAs sequences are evolutionarily conserved not only in mammals but also in the more evolutionarily distant *Drosophila* (Zhao B. et al., 2021; Mao X. et al., 2021). In addition, the expression of the same circRNA can vary greatly over time or in different tissues, as well as in diseased and non-diseased tissues (He A. T. et al., 2021; Verduci et al., 2021). Therefore, circRNAs have the potential to become good diagnostic markers for diseases and therapeutic targets.

### Biological Functions of circRNA

CircRNAs are involved in a variety of biological processes by acting as competing endogenous RNAs (ceRNAs), binding RBPs, regulating parental gene expression, and translating proteins or polypeptides.

## Acts as a Competitive Endogenous RNA

CircRNA contains a miRNA response element (MER), which can act as ceRNA to affect gene expression by competitively binding miRNA sites and inhibiting the regulatory effect of miRNA on downstream genes (Cheng et al., 2019; Hong et al., 2020; Luo et al., 2020; Su and Lv, 2020; Wang et al., 2021c). For example, Circ-CD44 is highly expressed in triple-negative breast cancer (TNBC), and high expression of circ-CD44 predicts poor patient prognosis. Circ-CD44 promotes KRAS expression through adsorption of miR-502-5p, thereby promoting TNBC proliferation, migration, and invasion. Circ-SNX6 acts as a molecular “sponge” to attenuate the inhibitory effect of miR-1184 on its target gene GPCPD1, thereby increasing intracellular lysophosphatidic acid levels, ultimately promoting resistance to sunitinib in renal cell carcinoma cells (Huang et al., 2021).

### Binding RNA Binding Protein (RBP)

CircRNAs can bind directly to RBPs to form RNA-protein complexes to regulate RBPs and further affect the expression and biology of downstream proteins (Huang et al., 2020; Feng J. et al., 2021; Chen J. et al., 2021; Xu et al., 2021). Muscleblind-like (MBL) binds to exon 2 of its parental gene and induces cyclization to form circMbl, which also binds to MBL to reduce MBL abundance, thereby reducing circMbl production (Ashwal-Fluss et al., 2014). Circ-hHBB3 binds HuR and degrades HuR, whereas Circ-TNPO3 competitively binds IGF2BP3 and inhibits the proliferation and metastasis of gastric cancer by regulating the MYC-SNAIL axis, which leads to malignant progression of GC (Yu et al., 2021). Circ-ACTN4 binds YBX1 and stimulates FZD7 transcription, which in turn promotes intrahepatic cholangiocarcinoma (ICC) proliferation and metastasis, leading to malignant tumor growth and metastasis (Chen et al., 2021c).

### Regulation of Parental Gene Expression

Some circRNAs also regulate the transcription of parental genes. Although most circRNAs are located in the cytoplasm, some circRNAs such as ElciRNA are in the nucleus of eukaryotic cells and play an important role at the transcriptional level (Humphreys et al., 2019; Ma N. et al., 2021; Greene et al., 2021). ElciRNA in the nucleus can interact with the U1 small nuclear ribonucleoprotein particle (snRNP) to contribute to the transcription of its parental genes (Song et al., 2016; Nan et al., 2019)and with the RNA-RNA of U1snRNA to enhance the cis expression of parental genes (Humphreys et al., 2019; Chu et al., 2021). In addition, a fraction of ElciRNAs accumulates in regions outside the nuclear transcription site, suggesting that this fraction may regulate transcription by acting in a trans manner (Ma J. et al., 2021). Introns derived from the Tulp4 gene can be cyclized to form circTulp4, which can interact with U1 snRNP and RNA polymerase II to regulate the transcription of its parental gene, Tulp4, thus participate in the development of Alzheimer’s disease (AD) (Ma N. et al., 2021). In summary, circRNAs can regulate parental gene expression through transcriptional regulation, splicing regulation, ceRNA, mRNA trap, translational regulation, and post-translational regulation pathways to regulate parental gene expression (Shao T. et al., 2021).

### Translation of Proteins or Peptides

Most circRNAs generated by reverse splicing are found mainly in the cytoplasm, and translation of linear mRNAs usually requires the structure of a 5′ end cap and a 3′ end poly(A) tail to remain stable. CircRNAs are not normally considered to have a translational function (Marquez-Molins et al., 2021; Yan and Bu, 2021) but they can initiate translation in the cell if they have an internal ribosome entry site (IRES) (He L. et al., 2021; Wang et al., 2021b; Sinha et al., 2021). This approach provides more polypeptide sequences and increases the polypeptide yield because the ribosome does not need to bind to RNA template repeats (Li P. et al., 2021). The cyclic RNA hsa-circ-0000437 can encode a functional peptide called CORO1C-47aa, and overexpression of CORO1C-47aa inhibits endothelial cell proliferation, migration, and differentiation by competing with the transcription factor TACC3 to bind ARNT and inhibit VEGF, which in turn leads to malignant progression of endometrial cancer (Li F. et al., 2021). Circ-RNA circ-FBXW7 encodes the FBXW7-185aa protein that inhibits the proliferation and migratory capacity of TNBC cells by increasing the abundance of FBXW7 and inducing c-Myc degradation (Ye et al., 2019). Circ-FNDC3B encodes a novel protein circFNDC3B-218aa and circFNDC3B-218aa significantly inhibits the proliferation, invasion, and migration of CC by suppressing the expression of Snail and promoting the expression of FBP1 (Pan et al., 2020). The translation of circRNAs and their regulatory role in tumor tissues are likely to provide new ideas for the study of circRNAs.

### M6A Methylation Modification Affects the Translation Ability and Nuclear Localization of circRNAs

M6A methylation modification is the most common modification method in eukaryotic RNA, and it mainly affects mRNA splicing, nucleation and translation. Recent studies show that modification of m6A can affect the nuclear localization of circRNA, and circRNA can also bind to the corresponding regulatory protein of m6A to affect its stability. Internal ribosome entry site (IRES) can promote the initiation of circRNA translation, and circRNA with single or multiple sites of m6 A modification can also initiate translation through IRES, which indicates that the presence of m6 A modification may affect the process of circRNA translation (He and He, 2021). CircZNF609 contains an open reading frame, which can translate a segment of mRNA sequence into amino acids, and regulate the translation process by recognizing m6A methylation modification proteins such as YTHD (Legnini et al., 2017). CircNSUN2 can interact with the m6A reading protein YTHDC1 to promote its own export from the nucleus to the cytoplasm in an m6A-dependent manner, and promote nuclear localization. Besides, circNSUN2 can interact with IGF2BP2 and high mobility group protein A2 (HMGA2) combined to form RNA-protein ternary complex circNSUN2/IGF2BP2/HMGA2 in the cytoplasm, enhancing the stability of HMGA2 (Li B. et al., 2021).

## Roles and Significance of circRNAs in Pediatric Malignant Solid Tumors

Many circRNAs are aberrantly expressed in pediatric malignant solid tumors and regulate tumor development.(Table 1).The stability and tissue specificity of circRNAs suggest that they are potential novel biomarkers for the diagnosis and therapeutic targets of pediatric malignant solid tumors.

**TABLE 1:** Functional characterization of circular RNAs in pediatric malignant soild tumor.

Circular RNAs	Expression	Role	Function role	miRNAs	Related genes	References
Hepatoblastoma						
hsa_circ_0000594	Upregulated	Oncogene	Promote cell proliferation, invasion, and suppress cell apoptosis	miR-217	SIRT1	Song et al. (2019)
circ-STAT3	Upregulated	Oncogene	Promote cell growth, migration and stem-cell characteristics	miR-29a/b/c-3p	GLI2	Liu et al. (2020)
circ-HMGCS1	Upregulated	Oncogene	Promote cell proliferation and inhibit cell apoptosis	miR-503-5p	IGF-PI3K-Akt	Zhen et al. (2019)
Neuroblastoma						
circ-CUX1	Upregulated	Oncogene	Promote aerobic glycolysis, growth, and aggressiveness	-	EWSR1/MAZ	Li et al. (2019a)
hsa_circ_0132,817	Upregulated	Oncogene	Promote cell proliferation, migration, invasion and glycolysis	miR-432-5p	NOL4L	Fang et al. (2021)
circ-DGKB	Upregulated	Oncogene	Promote the proliferation, migration, invasion, and tumorigenesis	miR-873	GLI1	Yang et al. (2020)
circ-CUX1	Upregulated	Oncogene	Promote tumor progression and glycolysis	miR-338-3p	PHF20	Wang et al. (2021f)
circ-KIF2A	Upregulated	Oncogene	Promote cell proliferation, migration, invasion and glycolysis	miR-129-5p	PLK4	Yang et al. (2021)
Circ-CUX1	Upregulated	Oncogene	Promote cell proliferation, migration, invasion and glycolysis	miR-16-5p	DMRT2	Zhang et al. (2020)
Wilms tumors						
hsa_circ_0093740	Upregulated	Oncogene	Promote the growth, migration and metastasis	miR-136/145	DNMT3A	Cao et al. (2021)
circ-CDYL	Upregulated	Oncogene	Promote cell proliferation, migration, and invasion	miR-145-5p	TJP1	Zhou et al. (2021b)
Rhabdomyosarcoma						
circ-ZNF609	Upregulated	Oncogene	Promote cell cycle	-	*p*-AKT and pRb/Rb	Rossi et al. (2019)
circVAMP3	Upregulated	Oncogene	Promote cell cycle progression	-	AKT-related pathways	Rossi et al. (2021)
Lymphoma						
circ-LAMP1	Upregulated	Oncogene	Promote cell proliferation and inhibit cell apoptosis	miR-615-5p	DDR2	Deng et al. (2019)
circ-APC	Downregulated	Tumor suppressor	Inhibit cell proliferation and tumor growth	miR-888	APC and Wnt/β-catenin	Hu et al. (2019)
circ-CDYL	Upregulated	Oncogene	Promote cell proliferation	miR-129-5p	NOTCH1	Mei et al. (2019)
				miR-3163	FMR1	
				miR-4662a-5p	ABCB1	
				miR-101-3p	TWIST1	
				miR-186-5p	VEGFA	
circ-CFL1	Upregulated	Oncogene	Promote cell proliferation, migration and tumor growth	miR-107	HMGB1	Chen et al. (2020)
circ-OTUD7A	Upregulated	Oncogene	Promote cell proliferation, metastasis, inhibit cell cycle arrest and apoptosis	miR-431-5p	FOXP1	Liu et al. (2021b)
circ-NSUN2	Upregulated	Oncogene	Promote cell proliferation and invasion	-	HMGA1/Wnt signaling	Wang et al. (2021a)
Medulloblastoma						
circ-SKA3	Upregulated	Oncogene	Promote cell proliferation, migration, invasion and induced apoptosis and cell cycle arrest	miR-326	ID3	Zhao et al. (2021b)
circ-SKA3 and circ-DTL	Upregulated	Oncogene	Promote cell proliferation, migration, and invasion	-	SKA3 and DTL	Lv et al. (2018)
circ-SKA3	Upregulated	Oncogene	Promote cell proliferation, migration, invasion tumor growth and inhibit cell apoptosis	miR-383-5p	FOXM1	Wang et al. (2020)
Adrenocortical Carcinoma						
circ-CCAC1	Upregulated	Oncogene	Promote cell proliferation, migration, and invasion	miR-514a-5p	C22orf46	Li et al. (2020a)

### Hepatoblastoma

Hepatoblastoma (HB) is an embryonal malignancy originating in the liver and is the most common malignant solid tumor of the liver in childhood (Kalish et al., 2017; Munoz et al., 2019; Hager and Sergi, 2021). The incidence of HB has been on the rise in recent years, with a yearly increase of about 4%, and its growth rate far exceeds that of other childhood malignancies, making it one of the major malignancies endangering pediatric health (Calvisi and Solinas, 2020; Prochownik, 2021). Surgery and chemotherapy are the main clinical treatments for HB, with a 3-year survival rate of 72.73% and a 5-year survival rate of 50.0% (Lake et al., 2019; Yang et al., 2019). Currently, the pathogenesis of HB is not clear and may be associated with multiple adverse factors such as genetic factors, immune response, low birth weight, and chromosomal abnormalities (Cristobal et al., 2019; Chen H. et al., 2021).

Circ-STAT3 (hsa_circ_0043800) is upregulated in HB tissues and cells, and inhibition of circ-STAT3 significantly inhibits HB cell growth, migration, and stemness. Circ-STAT3 can act as ceRNA by binding miR-29a/b/c-3p to upregulate Gli2 and STAT3, while Gli2 can activate the transcription of circ_0043800. *In vivo* experiments showed that circ_0043800 promoted HB tumor growth by upregulating Gli2 and STAT3 (Liu et al., 2020). Circ-HMGCS1 expression was significantly increased in HB tissues, and HB patients with high expression of circ-HMGCS1 had reduced overall survival. *In vitro* experiments confirmed that knockdown of circ-HMGCS1 inhibited HB cell proliferation and induced apoptosis. Mechanistic studies revealed that circ-HMGCS1 regulates IGF2 and IGF1R expression by binding to miR-503-5p and affects the downstream PI3K-Akt signaling pathway to regulate HB cell proliferation and glutamine catabolism (Zhen et al., 2019). Hsa_circ_0000594 expression levels were significantly upregulated in HB tissues and correlated significantly with HB subtypes. Inhibition of hsa_circ_0000594 significantly suppressed the malignant phenotype of HB and bioinformatics analysis indicated that hsa_circ_0000594 may regulate SIRT1 expression by binding miR-217 (Song et al., 2019).

### Neuroblastoma

Neuroblastoma (NB) originates from primitive sympathetic ganglion cells (Fetahu and Taschner-Mandl, 2021) and is a common extracranial solid tumor that occurs almost exclusively in children. It is the third most common tumor in children after leukemia and brain tumors (Zafar et al., 2021). Thirty percent of NB tumors occur in the adrenal medulla, about 60% in the abdominal paravertebral ganglia, and the rest in the sympathetic ganglia of the chest, head, neck, and pelvis (Rozen and Shohet, 2021). Currently, many new targeted therapies and immunotherapies have emerged in addition to conventional radiotherapy for NB (Blavier et al., 2020; Bhoopathi et al., 2021; Stainczyk and Westermann, 2021). NB is heterogeneous, and approximately 85–90% of children with low- and intermediate-risk NB can be cured, while the survival rate of children with high-risk NB is less than 50% (Aravindan et al., 2020; Quinn et al., 2021). Children with high-risk NB remain refractory to cure after multiple intensive treatments, and more than 50% of children relapse, with a 5-year survival rate of approximately 40–50% (Brignole et al., 2021). Therefore, it is important to investigate the molecular mechanisms underlying the development of NB.

Circ-CUX1 binds to EWSR1 and promotes interaction with MAZ, leading to transactivation of MAZ and transcriptional alterations of CUX1 and other genes associated with tumor progression. The use of inhibitory peptides that block circ-CUX1-EWSR1 interaction or LV-sh-circ-CUX1 significantly inhibited aerobic glycolysis, growth, and invasiveness of NB cells, suggesting that the circ-CUX1/EWSR1/MAZ axis is a therapeutic target for aerobic glycolysis and NB progression (Li H. et al., 2019). CircRNA hsa_circ_0132817 expression was significantly increased in NB tissues and cell lines, and knockdown of hsa_circ_0132817 inhibited tumor growth *in vivo*. Mechanistic studies suggest that hsa_circ_0132817 can promote tumorigenesis in NB cells by upregulating NOL4L and acting as a sponge for miR-432-5p (Fang et al., 2021). Circ-DGKB (hsa_circ_0133,622) expression was upregulated in NB tissues compared to normal dorsal root ganglia and negatively correlated with survival in NB patients. Circ-DGKB overexpression promoted NB cell proliferation, migration, invasion, and tumorigenesis and reduced apoptosis, promoting NB progression by targeting the miR-873/GLI1 axis *in vitro* and *in vivo* (Yang et al., 2020). Circ-CUX1 and PHF20 are upregulated in NB tissues and cells, while miR-338-3p expression is significantly decreased. Mechanistic studies revealed that circ-CUX1 promotes PHF20 expression, thus NB cell progression and glycolysis by binding to miR-338-3p (Wang Y. et al., 2021). Circ-KIF2A levels were increased in NB tissue samples and cell lines, and inhibition of circ-KIF2A significantly inhibited NB cell proliferation, migration, invasion, and glycolysis. Mechanistic analysis showed that circ-KIF2A could positively regulate PLK4 expression through the sponge miR-129-5p (Yang et al., 2021). Circ-CUX1 promotes NB cell proliferation, migration, invasion, and glycolysis. MiR-16-5p is a direct target of circ-CUX1 and miR-16-5p overexpression-mediated effects in NB cells can be partially alleviated by introducing circ-CUX1 overexpression plasmids. Another study showed that circ-CUX1 accelerates the proliferation, migration, invasion, and glycolysis of NB cells by targeting the miR-16-5p/DMRT2 signaling cascade (Zhang et al., 2020).

### Wilms Tumors

Wilms tumor (WT) is the most common primary malignancy of the kidney in children, accounting for 90% of all renal malignancies (Hohenstein et al., 2015). The etiology of WT is unclear and may be related to mutations in genes that regulate normal embryonic development of the urogenital tract (Stock et al., 2002; Fukuzawa and Reeve, 2007). Most patients have a palpable abdominal mass as the first symptom, and some patients may present with symptoms of hematuria, fever, urinary tract infection, varicocele, and anemia (Zhang et al., 2014). Currently, WT is treated with a multidisciplinary combination of surgical, chemotherapy, radiotherapy, and targeted therapy, with an overall cure rate of approximately 90% (Anvar et al., 2019; Palmisani et al., 2021; Pelosi et al., 2021).

The expression of circ-CDYL is significantly downregulated in WT tissues compared to adjacent non-tumor tissues and upregulation of circ-CDYL inhibited cell proliferation, migration, and invasion. Circ-CDYL acts as a miRNA sponge reducing the expression of miR-145-5p and further upregulating TJP1 expression (Zhou R. et al., 2021). Cao et al. found that hsa_circ_0093740 expression was significantly increased in WT and inhibition of hsa_circ_0093740 significantly suppressed the proliferation and migration of WT by high-throughput microarray sequencing. Mechanistic studies revealed that the hsa_circ_0093740-miR-136/145-DNMT3A axis plays an important regulatory role in WT growth and metastasis (Cao et al., 2021).

### Rhabdomyosarcoma

Rhabdomyosarcoma (RMS) is a malignant tumor arising from embryonic mesenchymal tissue, accounting for 15% of solid tumors and 50% of soft tissue sarcomas in children (Arndt et al., 2018; Skapek et al., 2019). The diversity of clinical manifestations, the multiplicity of pathological changes, and the different sites of onset make RMS one of the most complex pediatric tumors (Ramadan et al., 2020; Zoroddu et al., 2021). Surgical resection, chemotherapy, and radiotherapy are the main treatments for RMS (van Erp et al., 2018; Frankart et al., 2021). With the continuous improvement of chemotherapy regimens, the survival rate of RMS patients has increased to 70–80% (Pappo and Dirksen, 2018; Mohammad et al., 2020). Despite aggressive treatment, the 5-year survival rate for patients with metastatic RMS is still only 30% (Kashi et al., 2015; Pal et al., 2019), therefore, there is a need to find new diagnostic treatments and therapies for RMS to improve the survival rate of RMS patients.

Rossi et al. found that circ-ZNF609 expression was significantly upregulated in biopsies of embryonic RMS (ERMS) and alveolar RMS (ARMS). Knockdown of circ-ZNF609 in ERMS cell lines inhibited the cell cycle and led to a strong reduction in *p*-Akt protein levels and altered pRb/Rb ratios. In contrast, the knockdown of circ-ZNF609 had no significant effect on ARMS-derived cells but the exact reason for this is unclear (Rossi et al., 2019). Circ-VAMP3 expression is significantly increased in ARMS cells, and knockdown of circVAMP3 regulates the CCNB1/CDK1 complex, which controls the G2/M checkpoint by promoting the expression of CDKN1A and WEE1, thus the AKK1 and CDK1 complexes, as well as downregulation of Akt and ERK1, thereby inhibiting the cell cycle (Rossi et al., 2021).

### Lymphoma

Lymphoma is a highly heterogeneous disease (Liu M. K. et al., 2021) with increasing morbidity and mortality rates worldwide and is currently treated mainly with conventional radiotherapy (Leslie, 2021). Although the use of rituximab has led to significant improvements in long-term survival in some lymphoma patients, the treatment of relapsed refractory lymphoma remains a challenge (Shao L. et al., 2021; Takiar and Phillips, 2021).

We examined the differentially expressed circRNAs in normal infant thymus and T-cell lymphoblastic lymphoma (T-LBL) and found that circ-LAMP1 was significantly increased in T-LBL. Mechanistic studies revealed that circ-LAMP1 promotes cell proliferation and inhibits apoptosis through the miR-615-5p/DDR2 signaling axis, which in turn leads to malignant progression of T-LBL (Deng et al., 2019). Circ-NSUN2 is aberrantly highly expressed in malignant lymphoma tissues and cell lines, and circ-NSUN2 inhibition reduces the proliferation and invasion of lymphoma cells. Mechanistic studies have demonstrated that circ-NSUN2 can promote lymphoma progression by affecting Wnt pathways through the regulation of HMGA1 (Wang et al., 2021a). The expression of circ-APC (hsa_circ_0127621) is decreased in diffuse large B-cell lymphoma (DLBCL) tissues, cell lines, and plasma by microarray assays. Etopic expression of circ-APC inhibited cell proliferation *in vitro* and tumor growth *in vivo*. Mechanistic studies revealed that circ-APC acts as a sponge for miR-888 in the cytoplasm to upregulate APC, whereas, in the nucleus, circ-APC binds to the APC promoter and recruits the DNA demethylase TET1, which transcriptionally upregulates APC, thereby inhibiting the typical Wnt/β-catenin signaling pathway by reducing the accumulation of *β*-catenin in the nucleus and the catenin signaling pathway by reducing the accumulation of *β*-catenin in the nucleus, retarding the growth of DLBCL (Hu et al., 2019). In DLBCL, circ-CFL1 directly binds to miR-107 reducing the inhibitory effect on the target gene HMGB1, which promotes enhanced cell migration and proliferation as well as tumor growth (Chen et al., 2020). Circ-OTUD7A is highly expressed in DLBCL, and knockdown of circ-OTUD7A inhibits DLBCL cell proliferation and metastasis, promoting cell cycle arrest and apoptosis. Mechanistic experiments showed that circ_OTUD7A uptakes miR-431-5p to promote FOXP1 expression (Liu W. et al., 2021). Circ-NSUN2 is aberrantly highly expressed in malignant lymphoma tissues and cell lines, and circ-NSUN2 inhibition can reduce lymphoma proliferation and invasion. Mechanistic results suggest that circ-NSUN2, regulated by the transcription factor NRF1, can promote lymphoma progression by stabilizing the HMGA1-activated Wnt pathway (Wang et al., 2021a).

### Medulloblastoma

Medulloblastoma (MB) is a highly malignant neuroepithelial tumor of the central nervous system and is a common solid tumor in children (Li M. et al., 2021; Wen and Hadden, 2021), particularly those under 10 years of age (Hammoud et al., 2020; Danilenko et al., 2021). MB is difficult to treat because of its rapid growth, incomplete surgical excision, and tendency to disseminate with the cerebrospinal fluid (Caimano et al., 2021). The current 5-year survival rate for MB treated with a combination of surgery, radiotherapy, and chemotherapy is 65%. With the improvement of treatment efficacy and prolongation of survival, the toxicities associated with treatment have received increasing attention (Audi et al., 2021), therefore, it is important to search for potential diagnostic markers and therapeutic targets for MB.

Circ-SKA3 expression is elevated in MB tissues and cells, and inhibition of circ-SKA3 significantly inhibits MB cell proliferation, migration, and invasion, inducing apoptosis and cell cycle arrest and circ-SKA3 knockdown inhibits MB growth *in vivo*. Mechanistic analysis suggests that circ-SKA3 directly targets miR-326 to increase ID3 expression (Zhao X. et al., 2021). Differential expression profiles of circRNAs in four normal cerebellum and 4 MB samples using a HiSeq sequencer showed that thirty-three circRNAs were differentially expressed in MB tissue, of which three were upregulated and thirty were downregulated. Upregulated circ-SKA3 and circ-DTL promoted proliferation, migration, and invasion *in vitro* by regulating the expression of host genes as verified by *in vitro* cellular assays, demonstrating that circ-SKA3 and circ-DTL are critical in tumorigenesis and the development of MB (Lv et al., 2018). CircSKA3 and FOXM1 expression levels in MB tissues were significantly elevated, while miR-383-5p expression levels were significantly decreased. CircSKA3 was shown to uptake miR-383-5p and promote the expression of FOXM1. *In vitro* cellular assays confirmed that circSKA3 silencing significantly inhibited cell proliferation, migration, and invasion, promoting MB cell apoptosis (Wang et al., 2020).

### Adrenocortical Carcinoma

Adrenocortical adenocarcinoma (ACC) is a malignant endocrine tumor (Georgantzoglou et al., 2021; Kiesewetter et al., 2021) that typically occurs between 0–10 years and 40–50 years, with a higher incidence in children and women (Domenech et al., 2021). Approximately 60–70% of ACC patients exhibit clinical symptoms due to hormonal excess but many patients still have non-significant clinical symptoms (Fay et al., 2014). Common endocrine symptoms include cortisolism, masculinization, or gynecomastia (Ettaieb et al., 2020). The prognosis of ACC is poor, and tumor grade, stage, and hypercortisolism are associated with prognosis (Libe, 2015). Surgical resection is the treatment of choice but postoperative tumor recurrence rates are high and overall survival rates are low (Bedrose et al., 2020), therefore, it is important to identify good early diagnostic markers and therapeutic targets for ACC. Li et al. found that circ-CCAC1 was overexpressed in ACC tissue samples and cell lines and was associated with a poor prognosis. Functional assays showed that circ-CCAC1 enhances C22orf46 expression through uptake of miR-514a-5p promoting ACC progression (Li W. et al., 2020) (Table2).

**TABLE 2:** The potential of circRNAs in the diagnosis and prognosis of pediatric malignant soild tumor.

Study	Sample size	circRNAs	Expression	Source	Expression (*p* value)	Diagnostic value AUC	Sample size	Prognostic value OS (*p* value)	References
Hepatoblastoma									
Zhen et al	(Normal:Tumor)	circ-HMGCS1	Up	Tissues	*p* < 0.001	0.8283	Tumor (n = 33)	*p* = 0.0497	Zhen et al. (2019)
	(37:37)								
Neuroblastoma									
Yang et al	(Normal:Tumor)	circ-DGKB	Up	Blood	*p* < 0.05	0.7778	Tumor (n = 33)	*p* = 0.0324	Yang et al. (2020)
	(10:30)								
Zhang et al	(Normal:Tumor)	circ-CUX1	Up	Tissues	*p* < 0.05	-	Tumor (n = 50)	*p* = 0.0120	Zhang et al. (2020)
	(50:50)								
Lymphoma									
Mei et al	(Normal:Tumor)	circ-CDYL	Up	Tissues	*p* < 0.001	0.856	Tumor (n = 18)	*p* = 0.595	Mei et al. (2019)
	(17:18)								
Adrenocortical Carcinoma									
Li et al	(Normal:Tumor)	circ-CCAC1	Up	Tissues	*p* < 0.01	-	Tumor (n = 48)	*p* = 0.006	Li et al. (2020a)
	(48:48)								

### CircRNAs as Biomarkers of Pediatric Malignant Solid Tumors

**FIGURE 2:**
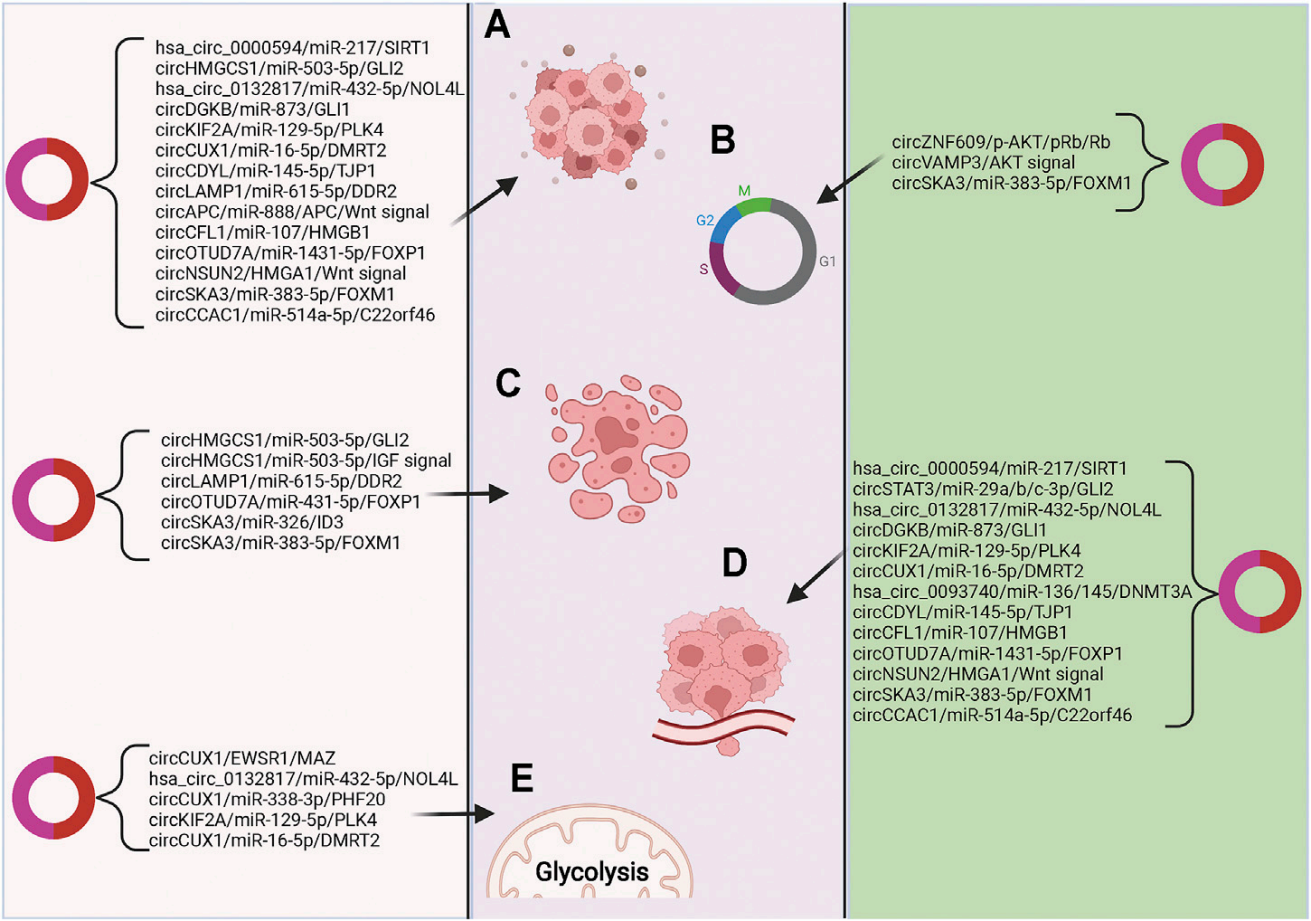
The relationships of circRNAs with pediatric malignant solid tumors **(A)** Signal pathways related to cell proliferation and differentiation; **(B)** Signaling pathways related to cell cycle regulation; **(C)** Signaling pathways related to apoptosis; **(D)** Signal pathways related to tumor cell invasion and metastasis; **(E)** Signaling pathways related to intracellular oxidative metabolism.

Many circRNAs that are highly expressed in blood have relatively low expression of their corresponding linear RNAs (Rahmati et al., 2021; Xiao et al., 2021). The unique functions and properties of circRNAs make them of great clinical potential in life sciences and medicine (Gu et al., 2021; Yi et al., 2021). Recently, many studies have explored the value of circRNAs for clinical application in pediatric malignant solid tumors, suggesting that some circRNAs have great clinical potential in early tumor diagnosis, treatment, and prognosis. (Figure 2).

Zheng et al. analyzed the association between circ-HMGCS1 expression levels and clinical features of HB finding a significant correlation between circ-HMGCS1 and AFP. The subject operating characteristic (ROC) curve was applied to investigate the diagnostic value of circ-HMGCS1 in distinguishing HB tissue from normative tissue showing that circ-HMGCS1 had diagnostic value (Area Under Curve (AUC) = 0.8283), with high expression of circ-HMGCS1 predicting poor prognosis in HB patients by Kaplan-Meier survival curve analysis (Zhen et al., 2019). The expression of circ-DGKB in blood was found to be of clinical importance in the diagnosis of NB by ROC analysis (AUC = 0.7778), with Kaplan-Meier analysis showing that patients with high levels of circ-DGKB expression had a lower survival rate (Yang et al., 2020). Zhang et al. divided NB patients into high and low circ-CUX1 expression groups according to the median value of circ-CUX1 expression for survival analysis revealing that high expression of circ-CUX1 in NB patients was associated with shorter survival times. In addition, high expression of circ-CUX1 was associated with advanced TNM stage, low differentiation grade, and positive lymph node metastasis in NB patients (Zhang et al., 2020). Mei and others reported that the plasma expression of circ-CDYL was significantly different between MCL patients and healthy controls (AUC = 0.856), with no statistically significant difference between circ-CDYL expression levels and prognosis of MCL patients (Mei et al., 2019). Li et al. found that high expression of circ-CCAC1 predicted poorer overall survival in ACC patients (Li W. et al., 2020).

## Conclusion and Future Perspectives

At present, there are few circRNAs studies in pediatric malignant solid tumors with variable results. However, all studies have shown that the expression of multiple circRNAs is dysregulated in pediatric malignant solid tumors, and some of the circRNAs target binding miRNAs that are involved in tumorigenesis and progression. The current exploration of tissue-specific circRNAs and studies of circRNAs-miRNAs-mRNAs networks have revealed the association of circRNAs with childhood malignant solid tumors. In addition, cellular and animal experiments have confirmed that for certain circRNAs that promote tumor growth, reducing their levels by targeted knockdown or RNA interference can inhibit further tumor growth and metastasis, suggesting that circRNAs are novel therapeutic approaches and drug targets for malignant tumors.

However, the main mechanism currently revolves around circRNA as ceRNA to regulate the function of tumor cells. Whether circRNA may affect the occurrence and progression of childhood solid tumors through other mechanisms The regulation is still unclear, and this needs to be further explored. In addition, most of the discoveries of circRNAs were not screened by next-generation sequencing technology, but were studied by referring to other articles, which lacked novelty. More importantly, only through high-throughput sequencing technology, the establishment of circRNAs expression profiles in childhood solid tumors is conducive to in-depth exploration of possible mechanisms.

Currently, the detection of circRNAs in tumors is mainly focused on tissue samples, which are more invasive and not suitable for early clinical tumor diagnosis (Zhou Q. et al., 2021; Lu et al., 2021; Shen et al., 2021) compared to other clinical samples such as serum, urine, and body fluids. CircRNAs are potential novel tumor biomarkers due to their high stability, conservatism, prevalence, tissue, and disease specificity (Ali et al., 2021; Tian et al., 2021; Vakhshiteh et al., 2021). Furthermore, the detection of free circRNAs in tumor tissues and the circulation of pediatric patients with malignant solid tumors is of great significance and value in terms of diagnostic accuracy, clinical staging, differential diagnosis, prognosis, and evaluation of treatment response. The identification of tumor biomarkers with high specificity and sensitivity among candidate circRNAs may improve the diagnostic accuracy and specificity of malignant solid tumors in children by combining them with other biomarkers or imaging examinations. In addition, it may also reduce the need for invasive procedures while helping to address the low organ specificity of existing tumor markers. At present, there are few studies on the clinical application of circRNAs in children with malignant solid tumors, and the sample size of included articles is also small, and there is a lack of multi-center studies. These are all problems that need to be solved urgently.

Moreover, the application of circRNAs as biomarkers for the diagnosis of malignant solid tumors in children is challenging. Although circRNAs are a promising biomarker due to their high organ-tissue specificity, current studies have not demonstrated that the sensitivity of candidate circRNAs is superior to that of known classical serum biomarkers. Compared to tissue biopsies, liquid biopsies have the advantages of non-invasiveness and reproducibility, however, circRNAs are low in body fluids, clinical data from peripheral blood studies are limited, and most circRNAs have not been shown to have excellent prognostic or diagnostic significance in large samples. Further studies should be conducted to evaluate the expression of circRNAs in serum and disease-related body fluids and there should be a consensus regarding sample handling, detection methods, and threshold values to enable the development of circRNAs as clinical diagnostic biomarkers. In addition, co-detection of tumors may lead to higher sensitivity and accuracy of diagnostic results.

In summary, although much is now known about the formation of circRNAs and their biological role and clinical application in childhood malignant solid tumors, the use of circRNAs for the treatment of pediatric malignant solid tumors requires further investigation. In addition, since many reviews on circRNAs in osteosarcoma have been published, osteosarcoma-related circRNAs were not included in the present review.
